# The pharmaceutics from the foreign empire: the molecular pharming of the prokaryotic staphylokinase in *Arabidopsis thaliana* plants

**DOI:** 10.1007/s11274-016-2070-z

**Published:** 2016-06-04

**Authors:** Katarzyna Hnatuszko-Konka, Piotr Łuchniak, Aneta Wiktorek-Smagur, Aneta Gerszberg, Tomasz Kowalczyk, Justyna Gatkowska, Andrzej K. Kononowicz

**Affiliations:** Department of Genetics, Plant Molecular Biology and Biotechnology, Faculty of Biology and Environmental Protection, University of Lodz, Banacha 12/16, 90-237 Lodz, Poland; Department for Good Laboratory Practice, Bureau for Chemical Substances, Dowborczykow 30/34, 90-019 Lodz, Poland; Department of Immunoparasitology, Faculty of Biology and Environmental Protection, University of Lodz, Banacha 12/16, 90-237 Lodz, Poland

**Keywords:** Molecular pharming, Plant expression cassette, Staphylokinase, *Agrobacterium*

## Abstract

Here, we present the application of microbiology and biotechnology for the production of recombinant pharmaceutical proteins in plant cells. To the best of our knowledge and belief it is one of few examples of the expression of the prokaryotic staphylokinase (SAK) in the eukaryotic system. Despite the tremendous progress made in the plant biotechnology, most of the heterologous proteins still accumulate to low concentrations in plant tissues. Therefore, the composition of expression cassettes to assure economically feasible level of protein production in plants remains crucial. The aim of our research was obtaining a high concentration of the bacterial anticoagulant factor—staphylokinase, in *Arabidopsis thaliana* seeds. The coding sequence of staphylokinase was placed under control of the *β*-*phaseolin* promoter and cloned between the signal sequence of the seed storage protein 2S2 and the carboxy-terminal KDEL signal sequence. The engineered binary vector pATAG-*sak* was introduced into *Arabidopsis thaliana* plants via *Agrobacterium tumefaciens*-mediated transformation. Analysis of the subsequent generations of *Arabidopsis* seeds revealed both presence of the *sak* and *nptII* transgenes, and the SAK protein. Moreover, a plasminogen activator activity of staphylokinase was observed in the protein extracts from seeds, while such a reaction was not observed in the leaf extracts showing seed-specific activity of the *β*-*phaseolin* promoter.

## Introduction

Due to outstanding advances made in the field of plant biotechnology, the way we look at plants and plant sciences nowadays has changed significantly. Plants are no longer considered only in terms of the diet consumed by humans and livestock or their natural values. Apart from animal cell cultures, yeast or bacterial production systems, plants have become one of the most promising alternatives for the synthesis of heterologous proteins. The phenomenon of the genetic transformation of plants and molecular pharming are now the best-known examples of modern plant biotechnology. Over the past decades a great number of proteins of important pharmaceutical or industrial value were produced in different plant species (Obembe et al. 2011). Leaves, tubers or seeds have become live bioreactors showing many advantages over the classic high-tech bioreactors and other biological production systems. Plants are less expensive to maintain requiring water, sunlight and standard agricultural input. Their multiplication through inbred seeds or vegetative propagation is well known. Moreover, there is no risk of contamination with human or animal pathogens (De Wilde et al. 2013; Lau and Sun 2009). But despite all these advantages and well developed technical background, this promising system of protein production needs to be improved. Usually two fundamental problems appear in connection with *in planta* transgene expression. Many of the heterologous proteins accumulate to low concentration or the process of their purification turns out to be more difficult and expensive than expected. To eliminate these problems, much efforts were made to engineer an optimal expression cassette—a set of strong regulatory sequences that would assure a high level of constitutive or time- or organ-specific synthesis of the active protein (Goossens et al. 1999; Van Droogenbroeck et al. 2007). Moreover, such a solution should make the protein purification and recovering processes easier and cheaper.

In this paper we present the research that tackles the common trends in modern plant biotechnology—the optimization of gene expression. Our idea of construction of the transgenic plants producing the recombinant SAK was inspired by the very interesting results achieved by the researchers from the University of Ghent. The research conducted by Dr. Depicker’s Group (published in *Nature Biotechnol.* in 2002) resulted in one of the highest protein accumulation levels ever reported for homozygous transgenic plant seeds—36.5 % of the total soluble protein (De Jaeger et al. 2002). This exceptionally high concentration was achieved through the use of the combination of the *β*-*phaseolin* promoter, arcelin5 5′-UTR, the *arc5*-*I*-*3′* flanking sequence and signal sequences.

The main goal of our work was optimization or finding the “know-how” key for the economically feasible synthesis of a recombinant protein—staphylokinase. Staphylokinase is a protein of bacterial origin showing plasminogen activator activity (Rooijakkers et al. 2005). It is a promising factor among other antithrombotic agents because the mechanism of its action is well-characterized (Rajamohan and Dikshit 2000) and to date, it remains one of the most fibrin-selective plasminogen activators known. Other currently used plasminogen activators (bacterial streptokinase, SK and hu-PA and ht-PA) lack fibrin specificity or they exhibit only a low one (Szarka et al. 1999). Cardiovascular diseases are one of the most common causes of death all over the world. Treatment of such diseases aims at slowing the formation of thrombi or breaking up the existing ones. In this respect, staphylokinase with its fibrin-selective PA activity appears to be almost ideal and is currently a topic of interest for scientists. To improve its natural properties, the new SAK variants are engineered (Chen et al. 2002, 2007; Szemraj et al. 2011). That is also why it was chosen as a model protein in this work. Initially, the recombinant staphylokinase was expressed in *Escherichia coli* or *Bacillus subtilis* (Behnke and Gerlach 1987; Gehmlich et al. 1997; Sako 1985). However, the protein purification process may appear to be more difficult, and thus economically less feasible than expected (Prasad et al. 2010). During the SAK recovery its activity may drastically decrease, for example due to the solubilisation process, thus both the bacterial and yeast systems for the staphylokinase production is still under investigation (Kotra et al. 2013; Moussa et al. 2012). Plants seemed to be an alternative and far more attractive system of staphylokinase production.

To achieve our goal, the recombinant shuttle vector was introduced to the *Arabidopsis thaliana* plants by the *Agrobacterium tumefaciens*–mediated transformation method. The plasmid was constructed on the basis of the pATAG5 vector combined with regulatory sequences harboured originally at the pPphas-G4 construct. The two fundamental elements of our vector—pATAG5 and pPphas-G4 plasmids—were kindly provided by Dr. Ann Depicker. Among others, because of dr. Depicker’s Group’s results, we decided to place the coding region of the *sak* gene under control of the *β*-*phaseolin* promoter. However, there were more reasons for the use of the regulatory elements from seeds. The high levels at which most of seed storage proteins accumulate make their regulatory sequences promising tools that we should take advantage of (Boothe et al. 2010). A seed-specific promoter that controls the expression of one of the most abundant protein in all *Phaseolus vulgaris* genotypes (phaseolin normally accounts for up to 50–60 % of the total protein) seemed to be an ideal candidate to achieve our goal (Chandrasekharan et al. 2003; De Jaeger et al. 2002; Goossens et al. 1999). The extremely effective activity of the *β*-*phaseolin* promoter probably results from its modular structure (Chandrasekharan et al. 2003). It possesses three TATA-boxes and is a subject to a stringent spatial and temporal-specific regulation (Emani and Hall 2008; Grace et al. 2004; Li et al. 2001). Over the past several years, several seed storage proteins and their regulatory elements were examined from the point of their possible application to the synthesis of the heterologous proteins in transgenic plants (De Wilde et al. 2013). The general conclusion was that the transcription and intron splicing processes occurred correctly and the introduced genes are expressed in a way similar to that in the species from which the regulatory elements were originally derived. Thus, it seemed very likely that the strong promoter of that kind could be freely used for the purpose of seed-specific expression even considering the heterologous nature of the host plant species. That is why it was also reasonable to use the *arcelin*-*5* expression signals for achieving the seed-specific expression of the *sak* transgene at sufficiently high levels in *Arabidopsis thaliana*. Like phaseolin, *arcelin*-*5* represents a family of very abundant proteins (30–40 % of the total seed protein content) (Goossens et al. 1999). The application of the mentioned sequences aimed at the developing of a winning transgenic plant system for the high level production of rSAK. However, the additional question was avoiding non-specific protein modifications and the problem of purification of the heterologous product. That is why the *sak* sequence was cloned between the amino-terminal signal sequence of the seed storage protein 2S2—for targeting at the ER and the carboxy-terminal KDEL sequence. The use of these two signal sequences constitutes the strategy of localized production of SAK. In the past, attempts of recovering pure staphylokinase from both plant cytoplasm and the cytoplasm of *E. coli* or *B. subtilis* had limited success since most of the bacterial therapeutics are produced in the form of inclusion bodies (Kotra et al. 2013). To avoid such difficulties and to assure appropriate posttranslational folding in plant cells, the SAK sequence was supplemented with the *ss* and KDEL motifs. After targeting at the ER (due to *ss* signal), SAK should reside in the endoplasmic reticulum channels. It should anchor at the ER membrane using its KDEL motif—a tetrapeptide conserved through mammals, plants, and yeasts responsible for the protein retention in the ER lumen (Xu et al. 2012). The foregoing solution had a lot of advantages. The ER seems to be the right compartment for the protein assembly and processing. There is a number of chaperone proteins inside and small amounts of proteases at the same time, which favours the formation of an active protein. It was reasonable to believe that under all those circumstances the level of expression of the recombinant SAK would meaningly increase.

## Materials and methods

### Material

*Escherichia coli* NM522 strain (Stratagene) was routinely used as a bacterial host for both: to multiply all the plasmid materials and for the subcloning of the intermediate products of the recombination. The engineered vector was introduced to the plant cells via *Agrobacterium tumefaciens*-mediated transformation (GV3101 strain, kindly provided by professor Szopa-Skórkowski from the Department of Genetic Biochemistry, University of Wrocław). *Arabidopsis thaliana* (L.) Heynh (Columbia genotype O) plants were used as a target plant host (the seed source: NASC The European Arabidopsis Stock Centre).

*Plasmids multiplied and maintained in E. coli*: pCAMBIA1304-*sak* (transformants selected under kanamycin selection pressure—50 μg/mL), pATAG5 (transformants selected under streptomycin selection pressure—20 μg/mL), pPphas-G4 (transformants selected under ampicillin selection pressure—50 μg/mL) and the recombinant vector pATAG-*sak* (transformants selected under streptomycin selection pressure—20 μg/mL). Both pATAG5 and pPphas-G4 plasmids were originally constructed and supplied by Dr Depicker’ team (De Jaeger et al. 2002), while pCAMBIA1304 vector (GenBank: AF234300.1) was obtained from commercial sources (http://www.cambia.org/daisy/cambialabs/materials.html) and then reengineered in our laboratory. The sequence of staphylokinase came from bacteriophage 42D (clone pDB17), GenBank: M57455.1 (http://www.ncbi.nlm.nih.gov/nuccore/M57455).

*Plasmids multiplied and maintained in A. tumefaciens*: the recombinant shuttle vector pATAG-*sak* (transformants selected under streptomycin, rifampicin and gentamicin selection pressure—in concentrations of 100, 25 and 10μg/mL respectively)

## Experimental protocols

### Plasmid construction

A shuttle vector pATAG-*sak* carrying staphylokinase structural gene was engineered using a combination of different regulatory elements. The process of construction consisted of several fundamental steps: *multiplication* of material, the *correctness verification* of the cloned substrates, *restriction digestion* of the inserts (*sak*-KDEL and the *β*-*phaseolin* promoter) and the destination vector (pATAG5), ternary *ligation* process and subsequent *transformation* into competent *E. coli* cells.

After subcloning of the required plasmids, the multiplication of the staphylokinase gene was carried out. The coding sequence of SAK was amplified by polymerase chain reaction (PCR) from pCAMBIA 1304-*sak* vector as a template. Through amplification two additional sequences were fused to the SAK gene in the form of primer sequences: a C-terminal sequence of the KDEL tetrapeptide and an *Xba*I restriction enzyme site. Due to the added cleavage site and *Nco*I restriction site staphylokinase was cloned into pATAG5 downstream from the *β*-*phaseolin* promoter, arcelin5 5′-UTR and 2S2 signal, followed by KDEL and the *arc5*-*I*-*3′* flanking sequences. The second element of the construct—the *β*-*phaseolin* promoter and its 3′ adjacent sequences (*arcelin*-*5* 5′-UTR and 2S2 signal) were cut out using the *Xho*I and *Nco*I restriction enzymes. However, it turned out that there was additional cleavage site for the *Nco*I restrictase within the promoter region. In that case, the optimal digestion time had to be established to obtain the full length target fragment. After the digestion products were electrophoretically separated in 1 % agarose gel, the promoter sequence was eluted from LMP agarose. Thus, the ligation solution included three DNA components digested with the appropriate enzymes: the *Nco*I ← *sak*-KDEL → *Xba*I fragment, the *Xba*I ← pATAG5 → *Sal*I linearized plasmid and the *Xho*I ← *β*-*phaseolin* promoter-2S2 → *Nco*I, mixed at the various ratios. All the reaction mixture variants were incubated overnight at room temperature. After the inactivation of the T4 ligase enzyme (Fermentas) at 65 °C for 20 min, the ligation solution was introduced to *E. coli* cells via the freeze and thaw transformation method. The selection process was run on a standard bacterial LB medium solidified with 1.5 % agar. Under the selective pressure, different numbers of streptomycin-resistant transformant colonies were obtained, depending on the ligation variants. It should be pointed out that one ligation event during the recombinant vector reconstruction was connected with the binding of sticky ends formed after cleavage with the use of different enzymes: *Sal*I and *Xho*I. After the pATAG-*sak* plasmids were isolated, the correctness of their structure was verified by molecular techniques like: PCR, restriction enzyme digestion and sequencing of the SAK gene and its short adjacent regions.

### *Agrobacterium tumefaciens* electrotransformation

The engineered vector pATAG5-*sak* was transformed subsequently into competent *Agrobacterium tumefaciens* cells (strain GV3101) using the standard electroporation method. For the electroporation, cells were grown to the mid-log phase and then washed extensively with water and 10 % glycerol. To electroporate DNA into bacteria, washed cells were mixed with the DNA to be transformed and then pipetted into a plastic cuvette. Each particular variant of electrotransformation had different voltage values and a different amount of plasmid DNA introduced. Electroporation was carried out using an ECM 399 Pulser (BTX Genetronics, Inc.) by applying a single electrical pulse of 1.44 or 2.2 kV with the apparatus set at 2.5 μF, 5 ms and 200 Ω. After receiving the pulse, the cells were suspended in 1 ml of the LB medium, transferred to culture tubes and incubated in the dark for 30 min at 4 °C. Then, the cells were left for 2 h at 28 °C with shaking (180 rpm). After the growth the putative transformants were spread on the selective media. Colonies arose from transformed cells after 2 days of incubation. The selection process was performed under the standard growth conditions on the bacterial YEB medium (Sambrook et al. 1989) solidified with 1.5 % agar and containing streptomycin, gentamicin and rifampicin. The presence and structure of *sak* transgene in the *Agrobacterium* cells were confirmed by the restriction analysis and PCR. The resulting recombinant strains were successfully used for *Arabidopsis* transformation.

### The conditions of the *Arabidopsis thaliana* cultivation and *Agrobacterium tumefaciens*-mediated transformation

After 2 weeks vernalization at 4 °C, *Arabidopsis* seeds were placed in small pots with the soil-perlite mixture (3:1, v/v). Plants were grown for 2 weeks in an 8-h light (at 22–23 °C)/16-h (at 20 °C) dark cycle. Then, the plants were bedded in separate pots and once the proper rosette had been shaped, they were bred in a 16-h light (at 22–23 °C)/8-h (at 20 °C) dark cycle. The first flowering shoot was removed to stimulate the secondary buds formation. Secondary shoots were subjected to the flower dip *Agrobacterium*-mediated transformation with recombinant *Agrobacterium* carrying the shuttle vector pATAG5-*sak* (Wiktorek-Smagur et al. 2009). The *Arabidopsis* shoots were dipped into the *Agrobacterium* suspension (OD_600_ = 0.8–0.9) supplemented with 300–350 µL Sillwet-77 (LEHLE SEEDS) for 2 min. Then, the plants were co-cultivated with the GV3101 strain for 20–22 h and then allowed to grow to maturity in the growth-room under controlled conditions of light, humidity and temperature. Finally, the seeds were harvested, left to dry for 2 weeks and then stored at 4 °C.

### Seed sterilization and antibiotic selection of transgenic *A. thaliana* plants

For selection of transgenic lines, the putative transformed seeds were surface-sterilized by a quick wash of 96 % ethanol, and transferred to a mixture of a commercial bleach containing 5 % sodium hypochlorite and 0.1 % Triton X-100 for 10 min. Then the seeds were rinsed at least four times with distilled H_2_O. Sterilized seeds were suspended in 0.1 % melted agarose and evenly spread on a half-strength MS medium (Murashige and Skoog 1962) supplemented with an appropriate antibiotic. Plants were grown at 22 °C under the same growth-room conditions used for the earlier tissue culture procedures in Petri dishes sealed with gas-permeable medical tape. At the stage of four leaves formed, the putative transgenic seedlings were individually bedded in an enriched selective medium (1 × MS, 1 % sucrose, 0.8 % agar) in small glass tubes. After the establishment of roots, the 2 week seedlings were transferred to pots with soil:perlite mixture and allowed to grow to maturity. For the first 3–4 days the pots were covered with a transparent plastic dome to protect the seedlings from low humidity. Transgenic seeds were then grown for the next generation to generate T_3_ seeds and analyzed. Transgenic seedlings (T_1_ generation) were selected on a growth medium containing kanamycin (Sigma) in concentration of 40 μg/mL. The T_2_ segregation was conducted and analyzed under the same conditions.

### Detection of the *sak* and *nptII* transgenes in selected *A. thaliana* plants

To confirm that the T-DNA of the shuttle vector pATAG5-*sak* was successfully integrated into the genome of transgenic plants, nested PCR analysis was carried out. The genomic DNA was isolated from T_1_ and T_2_*Arabidopsis* plants by the Dellaport’s method (1983), modified in The Faculty of General Genetics, Molecular Biology and Plant Biotechnology at the University of Lodz.

The staphylokinase gene was amplified using the following primers:

- 5′-AGG AGG AAA GGT ATA AGC AGG GG-3
- 5′-GGT GCC TAA TGA GTG AGA AAT TG-3.

The neomycin phosphotransferase gene was amplified using the following primers:

- 5′-GGA TCT CCT GTC ATC TCA CC-3
- 5′-CCA TGA TAT TCG GCA AGC-3.

### Protein extraction

The analysis of the expression of the staphylokinase gene was conducted on mature seeds harvested from transgenic T_1_ and T_2_*Arabidopsis* plants and from the wild type *A. thaliana* seeds for the negative control. To do that, 2 and 4 mg seed weighed amounts were frozen with liquid nitrogen and homogenized. Then, the ground seeds were extracted twice with hexane to remove lipids. Subsequently, the residues were lyophilized, extracted with an extraction buffer and then harvested by centrifugation (12,000 rpm, 4 °C). Total extractable seed protein samples were obtained using three different reaction buffers containing: **I**—20 mM Tris pH 8.0, 0.5 mM EDTA, 6 M urea; **II**—125 mM Tris–Cl, pH 6.8, 4 % SDS, 24 % glycerol, 10 % β-mercaptoethanol; **III**—50 mM Tris–HCl o pH—8.0, 5 mM EDTA o pH—8.0, 200 mM NaCl, 0.1 % Tween 20, a protease inhibitor mix (Sigma). The purified protein samples were stored at −80 °C. The protein concentration in the seed extracts was determined by the Lowry’s method (Bio-Rad Protein Assai Kit II).

### SDS-PAGE

The protein extracts were separated by SDS-PAGE using a *Mini*-*Protean* 3 apparatus (Bio-Rad). The electrophoresis was carried out in 12 and 15 % gels in a TGS buffer pH—8.3 (25 mM Tris, 250 mM Glicyna, 0.1 % SDS) at 120 V. The protein extracts were mixed with a buffer (125 mM Tris–Cl, pH 6.8, 4 % SDS, 24 % glycerol, 10 % β-mercaptoethanol, 0.02 % bromophenol blue) in the v/v ratio of 2–1. Then, the samples were incubated for 5 min at 95 °C. For the negative control the same amount of the wild type seed extract (SAK^-^) was prepared in the same manner.

### Preparation of specific rabbit anti-rSAK IgG antibodies

To obtain the specific immunoglobulins the laboratory New Zealand rabbit was immunized subcutaneously with 3 doses (200, 150, 150 µg) of commercial recombinant SAK (136 AA) obtained in *Escherichia coli* system (ProSpec) emulsified with the equal volume of complete Freund’s adjuvant (1st dose) (Sigma-Aldrich) or incomplete Freund’s adjuvant (2nd and 3rd doses) (Sigma-Aldrich) at 3-week intervals. Seven days after the last antigen boost the level of specific anti-rSAK IgG antibodies in a serum sample from the immunized rabbit was determined by ELISA with the homologous pre-immune serum serving as a negative control. The test was performed with rSAK as the coating antigen (at a final concentration of 1 µg/well) and rabbit serum samples diluted from 1:100 to 1:204,800 as a primary antibody. The immunoenzymatic reaction was developed using HRP-conjugated goat anti-rabbit IgG secondary antibodies (Jackson ImmunoResearch), H_2_O_2_ (Sigma-Aldrich) as a substrate and ABTS (Sigma-Aldrich) as a chromogen. The absorbance values were measured at λ = 405 nm. Since all serum dilutions were run in duplicate, the mean absorbance was calculated and the reactivity of the immune serum was determined by comparison to the cut-off value representing the mean OD value of the pre-immune serum at the concentration of 1:100 + 2 SD. The immune serum had IgG antibody titer higher than 1:204,800.

### Western blot analysis

Western blot analysis was performed according to the method described by Sambrook et al. (1989). Protein separation by SDS–PAGE was performed at 120 V. The proteins were overnight (30 V) transferred to a nitrocellulose membrane for immunoblotting. The primary antibodies were used at 1:5000 dilutions and the secondary antibodies (alkaline phosphatase-conjugated anti-mouse, Sigma-Aldrich, USA) were used at 1:2000 dilutions. Western blot analyses were performed using the Bio-Rad Western blotting apparatus (USA).

### Detection of the cofactor activity of staphylokinase in plant protein extracts

The plasminogen activator activity of SAK was analysed in both the leaf and seed protein extracts. The possible conversion plasminogen → plasmin (Plg → Plm) observed indirectly in the form of turning of the colour of the sample into yellow, would be evidence of the SAK presence. The yellow colour comes as a result of an enzymatic cleavage within the chromogenic substrate run by plasmin. The plasmin substrate used in these studies was H–D-Val-Leu-Lys-pNA S2251 (Chromogenix). The plasminogen activation assay involves a sequential reaction as follows:

1. PA + Plg → PA + Plm
2. PA + S2251 → pNA + H–D–Val–Leu–Lys–OH

The total assay volume was 200 μL. The reaction mixture included: 5 mM PBS buffer and 0.15 M NaCl, 1.1 µM human plasminogen (kindly provided by Professor J. Szemraj, Medical University of Lodz) and depending on the preparation—1.1 µM SAK (positive control) or the tested extract. The protein extract volume with the expected activation cofactor was added last to each cuvette to start the assay. For the negative control the wild type seed extract (SAK^-^) was used. After the 30–45 min incubation at 37 °C, the chromogenic substrate S2251 was added in the final concentration of 4 mM. The reactions were carried out at 37 °C in a microplate reader for 1.5 h. The change in absorbance was followed at 405 nm.

## Results

### Engineering of the shuttle vector pATAG5-*sak*

**Fig. 1:**
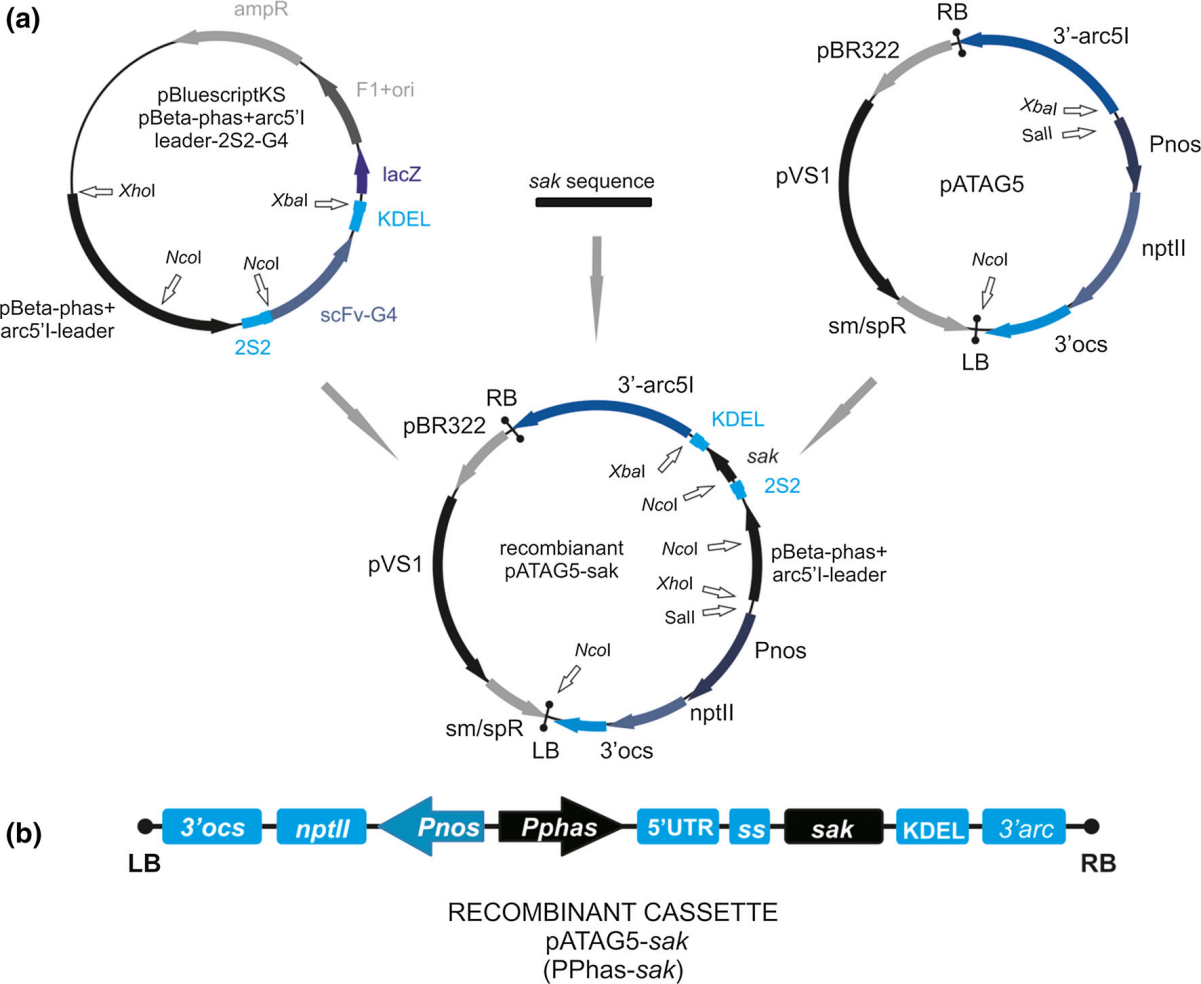
Outline of the pATAG5-*sak* construction process. The *sak* coding sequence and other regulatory elements from pßphas-G4 were cloned into the pATAG5 plasmid (**a**); Outline of the T-DNA region of the pATAG5-*sak*. Sequences: 3′ocs—3′ end of the octopine synthase gene; *nptII*—the neomycin phosphotransferase gene; *Pnos*—promoter of the nopaline synthase gene; *Pphas*—promoter of the beta-phaseolin gene; 5′UTR—5′ untranslated region of the *arc5*-I; *ss*—the coding sequence of the signal peptide from the 2S2 storage protein of *A. thaliana*; *sak*—staphylokinase gene; KDEL—the coding sequence of the carboxy-terminal ER retention signal; 3′arc—3′ flanking regulatory sequences of the *arc5*-I genomic clone; RB and LB, right and left border repeat of the T-DNA (**b**)

The expression cassette for the synthesis of the recombinant staphylokinase gene was engineered combining different regulatory sequences derived from three separate plasmids. All the DNA components were successfully cloned into the acceptor plasmid via ligation process, forming the binary plasmid pATAG5-*sak* (Fig. 1a, b).

The correctness of the engineered DNA molecule structure was confirmed by the PCR amplification and a series of restriction digestions, including these verifying the reconstruction of the restriction sites used for ligation. The final proof of the proper vector structure was provided by the DNA sequencing. The whole 2S2, staphylokinase and KDEL sequences and the *β*-*phaseolin* promoter and arc5-I 3′flanking region fragments were confirmed to be complete and in accordance with the sequences presented by their maps. After multiplication in *Escherichia coli* cells, the *pATAG5*-*sak* vector was used for the transformation of *Agrobacteriumtumefaciens*.

### Transformation of the *Arabidopsis thaliana* plants and T_1_ generation analysis

**Fig. 2:**
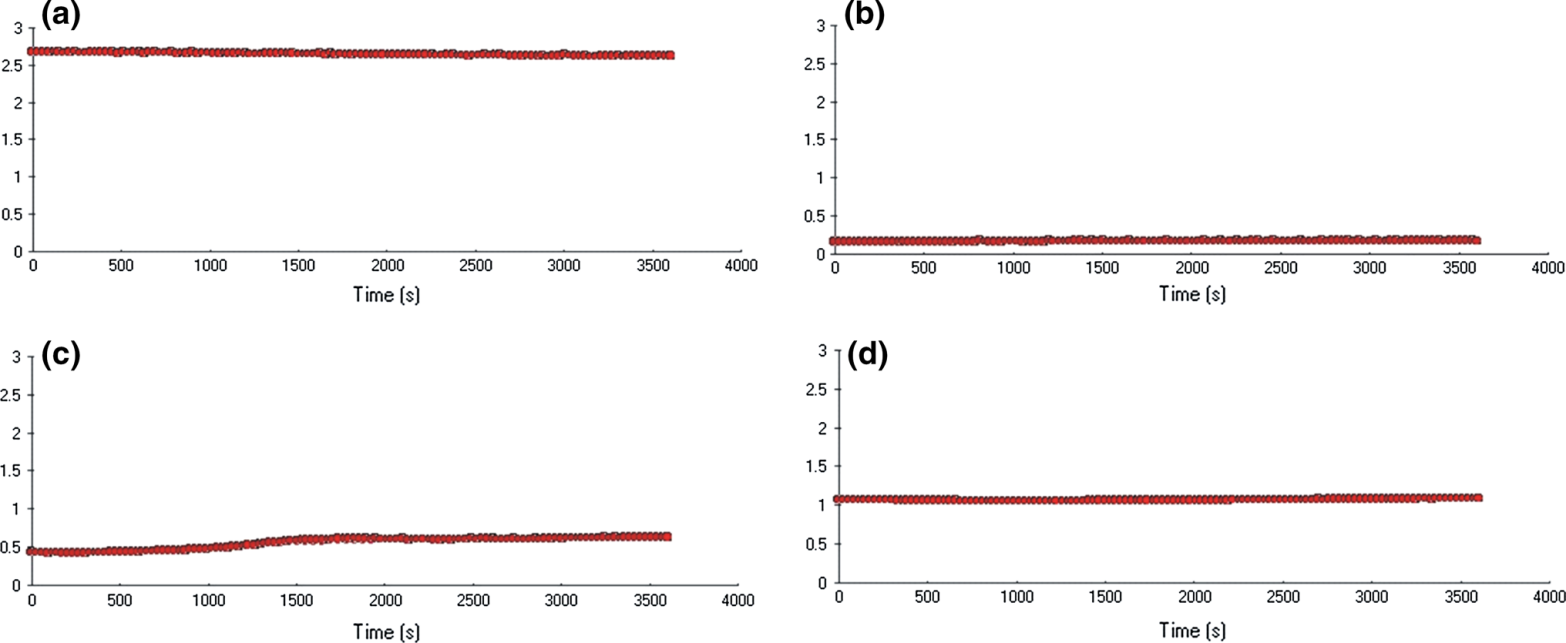
The amidolytic test results. Changes in the absorbance in the plant protein extracts: **a** positive control commercial SAK+; **b** negative control SAK−; **c** T1 seed extract; **d** leaf extract

For testing the activity of the expression cassette the expression construct was introduced into 13 *Arabidopsis thaliana* plants by an *Agrobacterium*-mediated transformation using the *floral dip* method. The first post-transformation seed stock (T_1_) yielded 17 putatively transgenic plants. They all were grown under the kanamycin selection pressure and were analysed by the nested PCR amplification of the *nptII* and *sak* gene fragments. Four of the tested plants turned out not to carry the transgenes (marked as the SAK− plants). It is not clear why they had survived on the selection medium, although the kanamycin concentration was consistent with the recommended one. All the selected plants were grown to maturity and the T_2_ seeds were collected. At this stage the initial analysis of the plant proteome was carried out. The total extractable protein samples were isolated at random from several seed harvests. Additionally, the total soluble proteins were isolated from the T_1_ leaves and the amidolytic test for staphylokinase activity was carried out. No activity was found in the leaf protein extracts, while some activity was observed in the samples of seed proteins. These results reflected tissue-specific activity of the *β*-*phaseolin* promoter (Fig. 2).

### Analysis of the T_2_*A. thaliana* plants

**Fig. 3:**
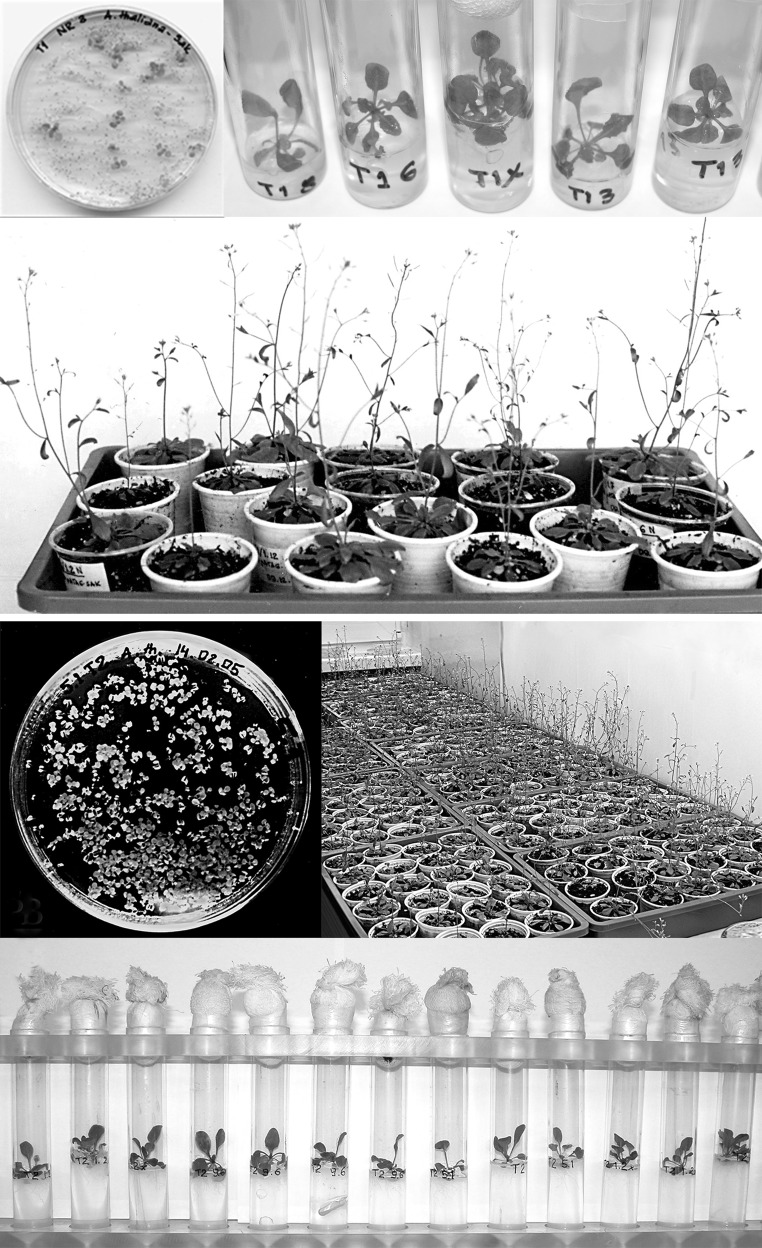
The process of the development of *Arabidopsis thaliana* plants. **a** T1 generation; **b** T2 generation. The first posttranslational seedstock (collected from 13 plants) yielded almost 600 plants, which makes generation enrichment extremely visible

All the selected *Arabidopsis thaliana* plants (13) went to seed, yielding almost 600 plants of T_2_ generation, which makes generation enrichment clearly visible (Fig. 3a, b).

**Fig. 4:**
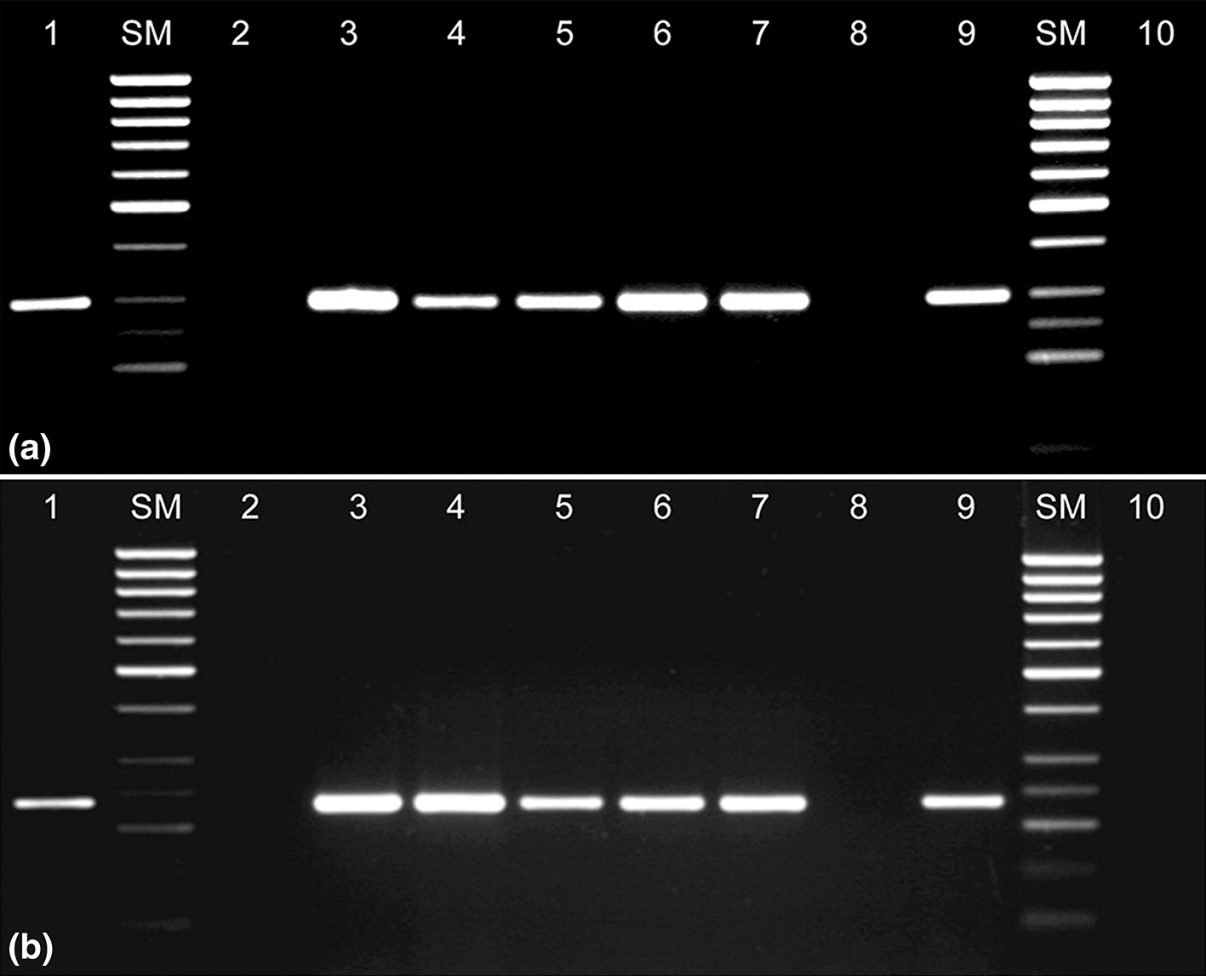
Results of PCR amplification. The genomic DNA isolated from T2 *Arabidopsis* plants was used as a template. **a** The *nptII* gene amplification (293 bp); **b** the *sak* gene amplification (221 bp)

To check whether the T-DNA fragments were successfully inherited, two nested PCR amplifications were carried out. It turned out that 78 % out of the 200 analysed plants carried both genes in question (*nptII* and *sak*) (Fig. 4a, b). The remaining unsuccessfully transformed plants (22 %) were marked as the SAK– plants.

**Fig. 5:**
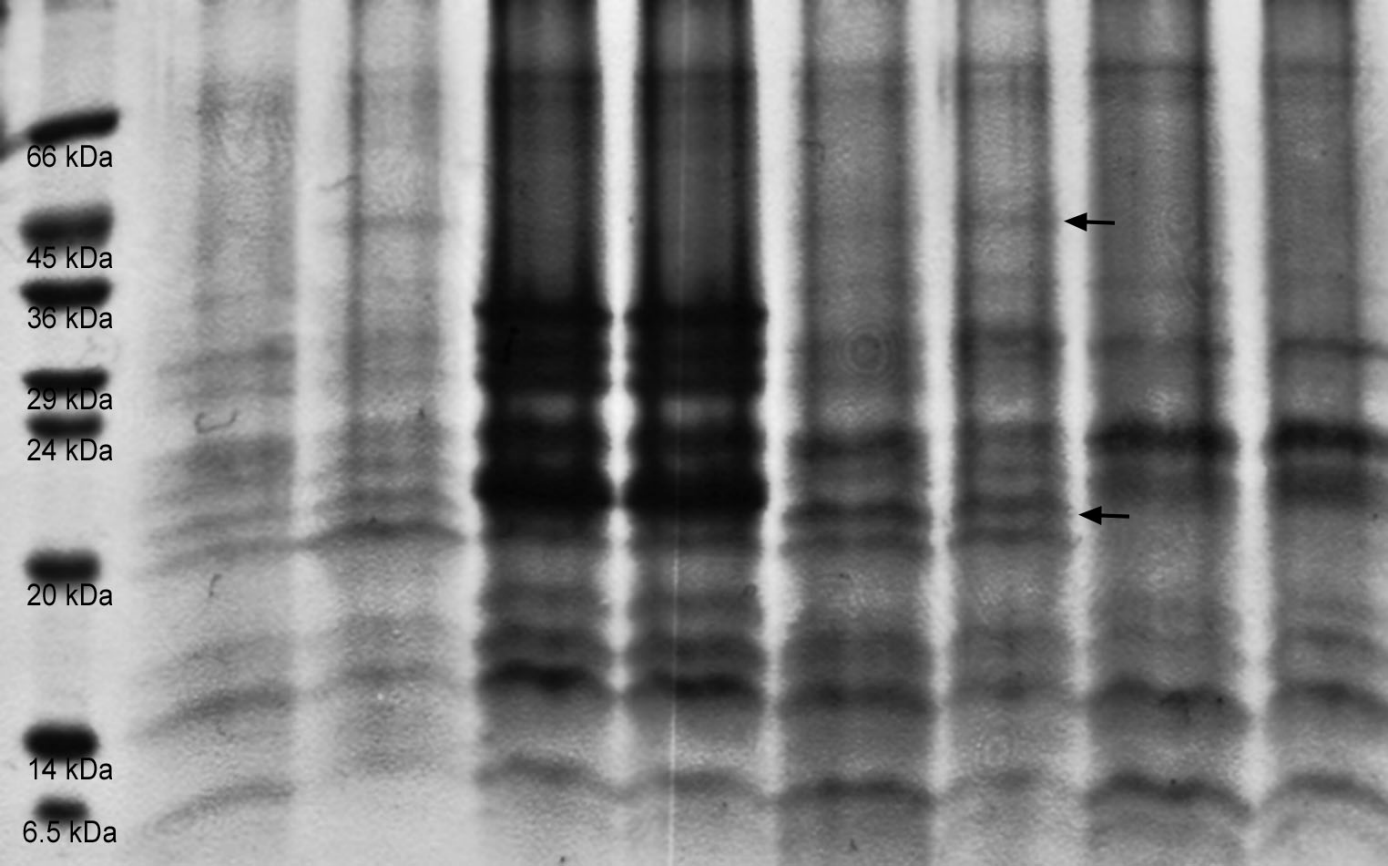
Results of protein separation in 12 % polyacrylamide gel. *Lane 1*—protein lader, SIGMA 6.5–66 kDa; No. M 3913; *Lane 2–7* seed protein extract SAK+; *Lane 8*—wild type seed protein extract (WT SAK−); *Lane 9*—Seed protein extract SAK−. The SAK monomers and dimmers/aggregates/fragments indicated by *arrows*

**Fig. 6:**
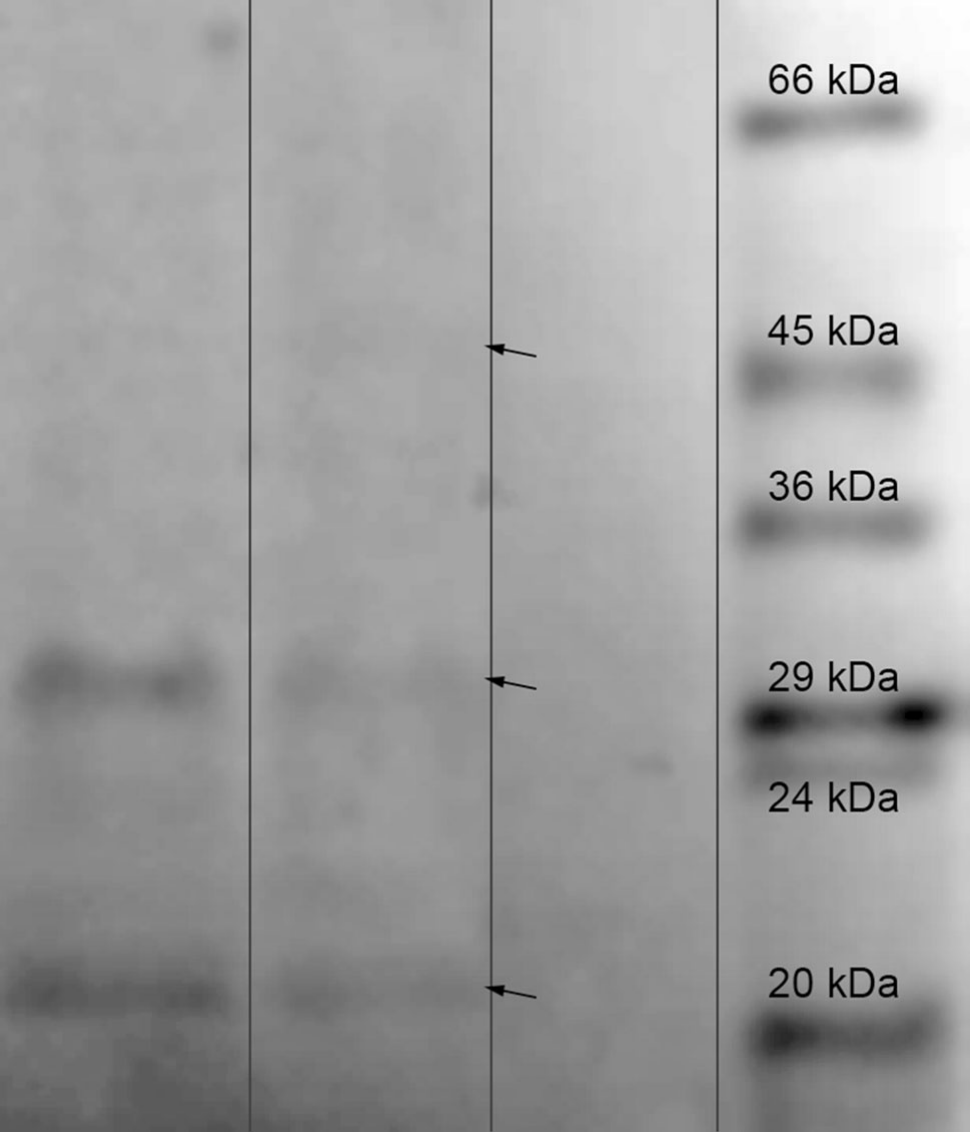
Results of Western blot analysis of the seed protein extracts. *Lanes 1, 2* protein extract SAK+; *Lane 3* protein extract from the wild type (SAK−) plants; *Lane 4* protein ladder (6.5–66 kDa, SIGMA). The SAK monomers (ca. 18.6 kDa) and dimmers/aggregates/fragments (ca. 30–45 kDa) indicated by *arrows*

To investigate the transgenic character of the proteome, the total soluble proteins were isolated from the T_2_ seeds. The total protein concentration in the seed extracts was determined by the Lowry’s method and its value ranged from 4.7 to 5.3 µg/µL. The proteins isolated with the use of three different buffers were separated by SDS-PAGE in 12 and 15 % polyacrylamide gels (data not shown). For the further analysis the protein samples extracted with the third buffer (50 mM Tris–HCl o pH—8.0, 5 mM EDTA o pH—8.0, 200 mM NaCl, 0.1 % Tween 20, protease inhibitor mix) were chosen. Bands (~20 kDa) corresponding to the molecular mass of staphylokinase (~18.6 kDa) were revealed in the 12 % gel. Such signals were not discovered in the lanes with the wild type SAK^−^ and SAK^−^ seed extracts (SAK negative controls) (Fig. 5). Similarly, this SDS-PAGE separation revealed additional slightly visible bands only in transgenic lanes corresponding to the mass of 45 kDa (SAK dimmers?), not observed in the wild-type SAK^−^ extract. Finally, Western-blot analysis of randomly chosen potentially transgenic plants yielded two main protein fractions, again not observed in the wild-type SAK− extract, which confirmed the SDS-PAGE results (Fig. 6). On the side note, it should be pointed out that Western blot analysis for unsuccessfully transformed SAK− plants were conducted as well, however, none of the staphylokinase-like proteins were detected (*figure not shown*). It is also important to say that in most cases the presence of SAK could not be determined on the SDS-PAGE gels (almost 460 plants analyzed). There were only over a dozen transgenic lines that could be indentified directly on the electrophoregrams and their content of staphylokinase was preliminary assessed in the SAK+ *Arabidopsis thaliana* plants via protein quantification using *ImageJ* software. Densitometric analysis revealed that the concentration of staphylokinase varied from 0.0 to 5.9 % of the TSP (*mean* ≈ 0.043 % for all tested plants, and from 0.42 to 5.9 % among the SDS-PAGE SAK+ plants).

To determine the cofactor staphylokinase activity in the total protein extracts, the amidolytic activity assay was carried out. Although the level of the SAK activity was far lower than expected, it should be pointed out that undoubtedly it was higher than this observed previously (during the T_1_ generation analysis). The amidolytic test revealed low staphylokinase activity in 13 out of 48 randomly investigated samples (Fig. 8). Such result provoked a question whether there were any native plant factors that inhibit the amidolytic activity of SAK. That would result in the low or no activity observed during the amidolytic test. To answer this question a test for inhibition of the commercial synthesized SAK was carried out (data not shown). However, even mixed with the plant protein extracts, the commercial rSAK exhibited activity comparable to this of the SAK in positive control preparation. Thus, the trace activity of the bacterial staphylokinase in the plant protein extract may have been caused by different reasons.

## Discussion

Today, diverse systems for the synthesis of the heterologous proteins are available. However, in most of them, high expression levels of correctly folded recombinant proteins are difficult to achieve (He et al. 2008). Transgenic plants seem to have the highest potential as cost-effective bioreactors for the production of foreign proteins at the economically significant level. The recent studies, resulting in the constantly increasing number of proteins produced in plant tissues, obviously support such a conclusion. However, recovering of recombinant proteins obtained in plants often appeared to be labor consuming and expensive. Thus, the designing of the optimal plant expression cassette to synthesize large quantities of active, easily purified proteins, makes one of the present-day approaches, as opposed to the “post expression” approaches focusing e.g. on the improvement of protein purification techniques (Kowalczyk et al. 2014).

In this paper we presented the results of the application of the seed storage protein regulatory sequences for the expression of the bacterial staphylokinase gene in plants. Considering the extremely effective synthesis and the ability for the accumulation of the seed storage polypeptides in the developing seeds, the use of their “expression control elements” was well justified. Consequently, so far, the analysis of the abundant protein expression pattern yielded the much efforts to transfer “the synthesis solutions” of the seed storage protein genes to a heterologous system. Our research could allow a preliminary assessment of the feasibility of the expression and purification of biologically active staphylokinase produced in plant seeds with the use of very promising regulatory elements. To realise this goal, we engineered the shuttle vector pATAG5-*sak* carrying the *sak* gene, cloned between the seed storage protein regulatory elements and provided with the ER localization signals. The construct was successfully introduced to the plant cells via *Agrobacterium tumefaciens*-mediated transformation. The results we present here were obtained in the model plant species *Arabidopsis thaliana*, which was quite advantageous considering the fact that we have certain experience in the field of staphylokinase synthesis in the *Arabidopsis* plants as well (Wiktorek-Smagur et al. 2011). Although previously the CaMV35S promoter had been used for the SAK synthesis (also in *Nicotiana tabacum* and *Solanum tuberosum*), the obtained results could be initially compared to each other and more effective production system could be indicated. So far, our attempts to produce SAK in plant tissues with the use of the CaMV35S promoter had limited success. Thus, the described research project makes a continuation of the studies over the recombinant staphylokinase production in plants conducted in our Department (Gerszberg et al. 2012). However, in the light of our past results, we decided to change the expression cassette components. Apart from our results, our decision about replacing the CaMV promoter with the alternative one was caused by other reasons. The same low protein concentration was reported by De Jaeger et al. (1999) during the scFv peptide synthesis with the use of the CaMV35S promoter in the *Petunia hybryda* tissues. Three years later, De Jaegera and coworkers (2002) confirmed the low activity of the virus promoter, obtaining the scFv accumulation at the level of 1 %. All these results made us look for an alternative promoter. Additionally, the tissue- or temporal-specific expression have become prevalent recently. But the most important fact is, that it was the *β*-*phaseolin* promoter that had yielded one of the highest level of the heterologous protein scFv—36.5 % (De Jaeger et al. 2002). Using the same cassette, Van Droogenbroeck and coworkers (2007) demonstrated the expression of a single-chain antibody fragment fused to an Fc fragment (scFv-Fc) at the level of 10 % of the TSP in *Arabidopsisthaliana* seeds. Apart from the promoter, the 5′UTR and 3′ flanking sequences were changed as well.

**Fig. 7:**
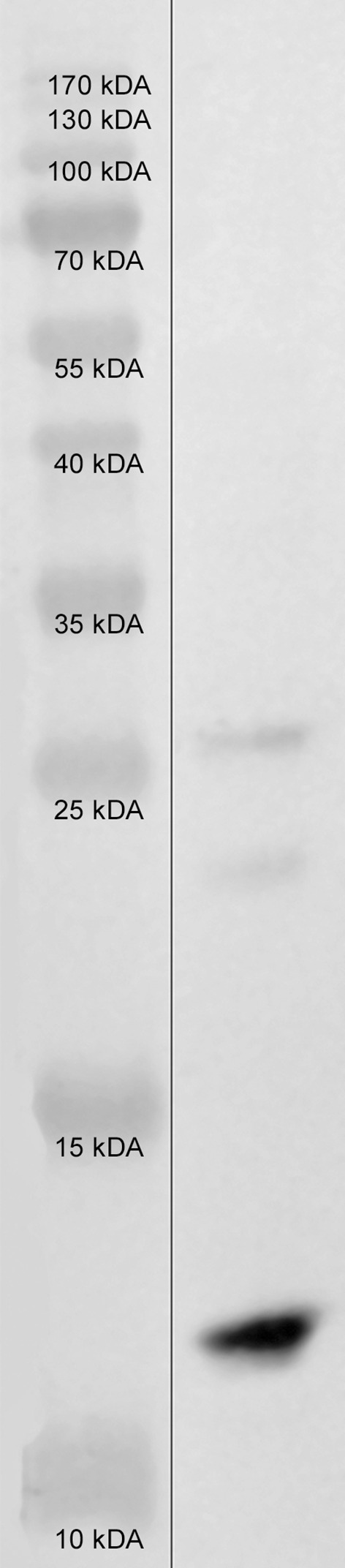
Results of Western blot analysis of the commercial recombinant SAK protein from *E. coli* system. *Lane 1* PageRuler Prestained Protein Ladder (10–170 kDa). *Lane 2* commercial recombinant SAK protein, 15.5 kDa (0.25 microgramme per lane)

Undoubtedly, the obtained results demonstrated that the chosen molecular strategy worked. The presence of SAK in the *Arabidopsis thaliana* plants is unquestionable, both at the genomic and proteomic levels. The direct proof for the expression of SAK in the eukaryotic system of plants was the Western-blot analysis. Similarly to SDS-PAGE analysis, we found several bands present on the blot-membrane. It seems likely that these are the SAK variants migrating at varied levels (SAK monomers—ca. 18.6 kDa, dimmers/aggregates/fragments—ca. 30–45 kDa), particularly since the commercial recombinant SAK isolated from the protein extract from *Escherichia coli* cells created a similar pattern of migration (Fig. 7). Moreover, it is no surprise since both the electrophoresis and immunodetection reactions were conducted using the total protein extracts from the extremely protein-rich seeds, where protein–protein interactions were unavoidable. Similarly, the tested extract contents (total seed protein extracts) may also cause slight differences in the migration level of recombinant monomers (SAK monomers mass is ca. 18.6 kDa and bands observed in the potential transformants migrate about 20 kDa). Hence, the presence of recombinant proteins in the investigated transformants seems to be beyond all doubts, especially since they were also tested for their plasminogen activator potential and displayed a positive response.

However, in spite of the complete change of the expression strategy, the staphylokinase synthesis turned out to be less effective than expected. Although the *β*-*phaseolin* promoter and the regulatory sequences of the seed storage protein *arcelin5*-*I* were used, the expression level of the staphylokinase still remained unsatisfactory. The expression level of the purified SAK was not determined, but the SAK-like bands analysis from SDS-PAGE revealed a low concentration of the tested recombinant, *mean* ≈ 0.043 % for all tested *Arabidopsis* plants (some higher concentrations, from 0.42 to 5.9 % among the SDS-PAGE SAK + plants, were also observed, however, they come from the assessment of the abundance rate of the SAK against the Total *extractable* Seed Protein isolated with a specific buffer from the SDS-PAGE SAK + plants, not directly comparable in our opinion). All this proves that it is still not easy to meet the economic demands and develop a novel effective expression system in plants. A similar, in a way, technology of *in planta* synthesis of heterologous proteins (insulin as a model protein) has been worked out by the researchers from the Calgary University (Nykiforuk et al. 2006). However, even the use of the *β*-*phaseolin* promoter did not benefit in the form of a significant accumulation increase—the authors reported that the insulin accumulated at the level of 0.13 % of the total seed protein. Anyway, there can be many reasons for such situation, as we did not determine the critical point at which the staphylokinase expression was inhibited. It should be pointed out that to the best of our knowledge and belief it is one of few examples of the expression of the prokaryotic protein in the eukaryotic system, and still the only example of the expression of staphylokinase in plants (apart from our previous studies). There are no other plant systems producing staphylokinase to confront our results. Perhaps the reason for the low concentration is of a quite different nature than the bacterial origin of the transgene. It may come, for example, from the difficulties during the purification process or it may result from the transient expression phenomenon. The last option would connect with the variation of the expression pattern in the subsequent generations. However, studying number of the plants of confirmed transgenic nature (78 % out of all T_2_ plants at the genome level), grown in the successive generations, the phenomenon of the generation enrichment seemed to be indisputable. It rather proves the stable nature of the integration. Considering the situation from the point of the expression cassette components, we can assume, that such concentration may result from the number of the copies of the regulatory sequences cloned into T-DNA fragment. Namely, it may be explained in part by the fact that seed storage protein genes belong to the multigene families. It seems very likely that an individual copy of all the regulatory elements will not assure as high an expression level as when the native number of copies is used. On the other hand, there could occur more than one T-DNA integration event. By contrast with the single or a few copy insertion, such an integration pattern may cause gene silencing. Gene silencing is one of the common reasons for the gene inactivation or the expression variation among eukaryotic cells (Meyer 1996; Kooter et al. 1999). The gene silencing may occur at various levels of gene expression, including the transcriptional or post-transcriptional stages (Fagard and Vaucheret 2000). Among the causes of the gene inactivation we can find a well-known methylation of cytosines, RNA virus activity, the presence of the aberrant RNA molecules, the presence of cryptic stop codons, false polyadenylation or splicing signals, the interactions between transgene repeats, or finally the position effect (the effect of the site a transgene is inserted in) (Matzke and Matzke 1998; Day et al. 2000; De Wilde et al. 2000; Butaye et al. 2005).

**Fig. 8:**
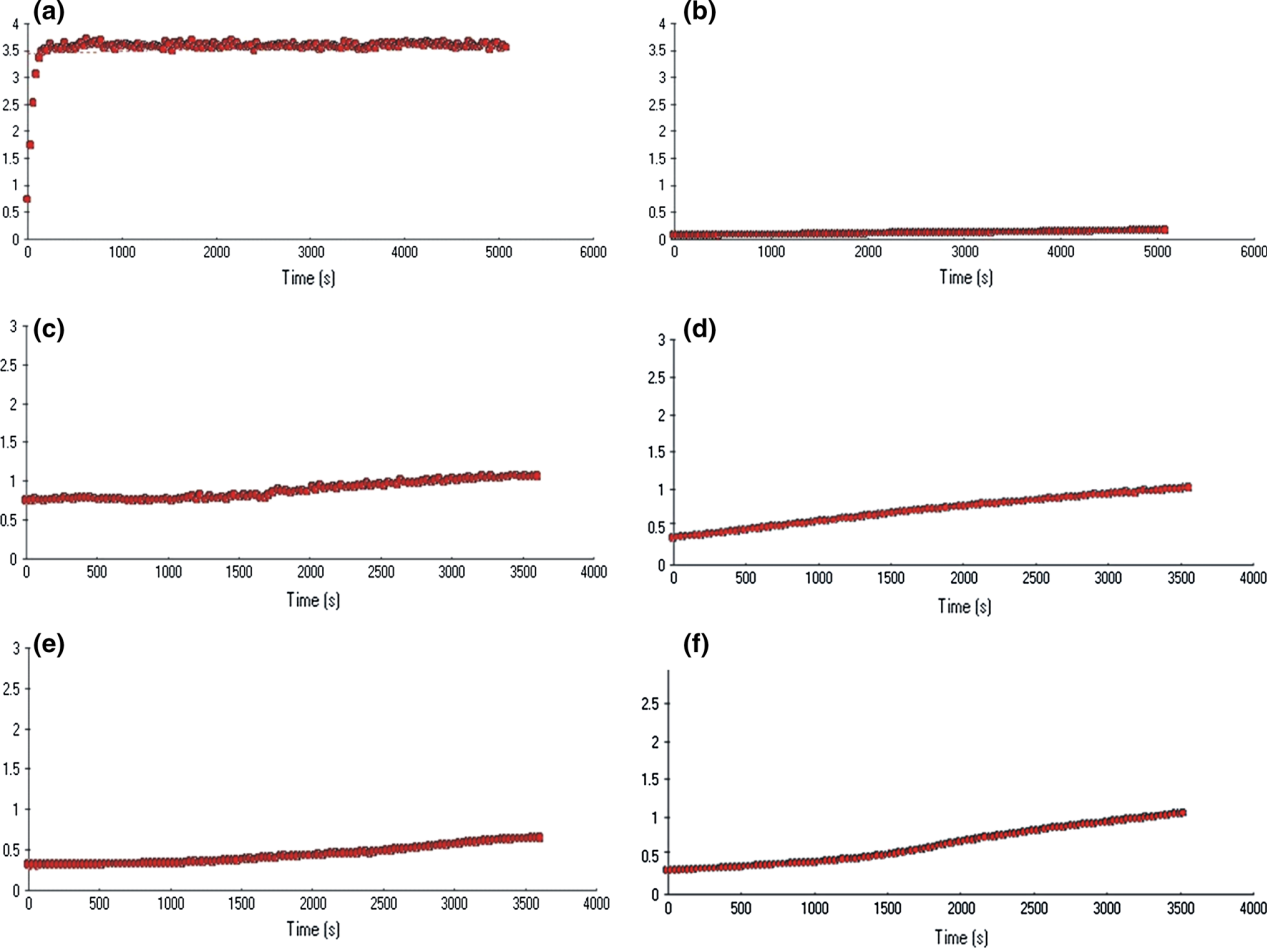
The amidolytic test results carried out in the T2 seed extracts. The changes in the absorbance in the plant protein extracts: **a** positive control, commercial SAK+; **b** negative control containing the wild type SAK− seed protein extract; **c**–**f** the investigated seed protein extracts

The presence of the staphylokinase in the *Arabidopsis* protein extracts was revealed also indirectly by its trace cofactor activity during the amidolytic activity assay, which makes the strongest argument for the successful transformation. The low concentration of the prokaryotic factor may be caused by the reasons listed above or it may result from the recovery difficulties. The latter hypothesis is supported by the electrophoretograms. The variable presence of the SAK-like bands in the lanes containing the same protein samples, but obtained after extraction in different buffers, indicates a diverse extraction capability. It would mean that the SAK molecules are synthesized in the plant cells, but they are recovered in altered forms and rather low concentrations. The results of the amidolytic activity assay carried out for the leaf and seed extracts are the strongest and most important evidence for this hypothesis. The trace level of the cofactor activity observed in the T_2_ seed extracts in comparison to the complete lack of the absorbance changes in the T_1_ leaf extracts confirms the seed-specific activity of the *β*-*phaseolin* promoter. The amidolytic analysis of the T_2_ generation revealed a stronger amidolytic activity and, indirectly, the higher SAK concentration (Fig. 8). The number of individual positive results increased as well (13 out of 48). In this case the purification procedure should be improved or *de novo* established. The ER-directed accumulation of the SAK has several advantages, e.g. the protection from non-specific posttranslational modifications. However the process of the protein recovery appeared to be very difficult, especially considering the fact, that staphylokinase could be anchored in the ER membrane. Its releasing required application of strong chemicals or a mechanical factor, which in turn could destroy the SAK structure. This together with the correct post-translational folding conditioned the biological activity of the protein. On the other hand, there is a possibility that the KDEL-tag may not only contribute to the ER localization but also regulate delivery to other compartments, hence varied patterns of deposition of the protein-tagged might be observed. Suggestions appeared indicating factors that might influence the ER retention: for example the amount of KDEL-tags per engineered molecule or the accessibility and integrity of the tag (De Meyer and Depicker 2014). If such an option was taken into account and our protein abandoned the ER structure, the question of non-specific SAK modifications would remain open (Frigerio et al. 2001). Thus, owing to the latter hypothesis, we would search for another reason for the low activity of staphylokinase, for example the glycosylation processes. Of course, apart from the mentioned scenario, the unsatisfactory level of the SAK synthesis could come from the exceptional reactivity of the bacterial protein. Consequently, staphylokinase may easily create self-aggregates or bind to different plant molecules, which in turn could be observed as spatial obstacles during the proper configuration formation. It is also possible that the interaction with other proteins could block some of the staphylokinase regions essential for the plasminogen activation.

In conclusion, the main goal of our research—the seed-specific expression of the recombinant staphylokinase in the *Arabidopsis thaliana* plants—was achieved. Since further research is still to be done, it is difficult to indicate the best expression system among these we were working over. At this stage both the CaMV35S and *β*-*phaseolin* promoter constructs have certain advantages and defects. Moreover, their comparison is not possible because the purified SAK expression level obtained with the use of the *β*-*phaseolin* promoter was not determined. The amidolytic activity assay was perceived only in terms of qualitative, not quantitative assessment. The recombinant SAK concentration and cofactor activity was too low to convert them into the SAK specific activity. However, in spite of this moderate success, our knowledge about the staphylokinase and the possible difficulties during its expression in the plant system sufficiently increased. We hope to make the best use of it in due course, while further attempts to achieve the prokaryotic protein expression in plant tissues will be taken.
